# Delayed Presentation of Metastatic Solid Pseudopapillary Epithelial Neoplasm in a Pregnant Woman: A Case Report

**DOI:** 10.7759/cureus.82454

**Published:** 2025-04-17

**Authors:** Amer Tamr, Muhammad Ali Shahid, Ira Wollner, Brian Theisen

**Affiliations:** 1 Internal Medicine, Henry Ford Health System, Detroit, USA; 2 Hematology and Oncology, Henry Ford Health System, Detroit, USA; 3 Pathology and Laboratory Medicine, Henry Ford Health System, Detroit, USA

**Keywords:** chemotherapy, hormone therapy, metastatic solid pseudopapillary epithelial neoplasm, pregnancy, surgical resection

## Abstract

Solid pseudopapillary epithelial neoplasm (SPEN) is a rare, low-grade malignant pancreatic tumor usually found incidentally on imaging, most commonly in young women. Its association with women suggests that hormones may play a significant role in tumor pathogenesis. Surgical resection is the mainstay of treatment in all stages of disease, and few other treatment options have been thoroughly explored. This case demonstrates the unique therapeutic challenges involved in the management of a pregnant woman with new liver metastasis and significant disease burden following remote resection of the primary SPEN tumor. The patient was treated with chemotherapy, radiation, and hormone therapy, followed by hepatic trisegmentectomy without recurrence during surveillance. This case presents a unique therapeutic approach to a situation where no established treatment guidelines exist.

## Introduction

Solid pseudopapillary epithelial neoplasm (SPEN) is a very rare, low-grade malignant pancreatic tumor. Patients typically present with nonspecific features, primarily nontender abdominal pain, but many patients are asymptomatic with lesions found incidentally on imaging [1]. It classically presents as a single, large, well-circumscribed lesion that appears cystic, solid, or mixed cystic and solid on imaging and is usually surrounded by a dense fibrous capsule [1,2]. SPEN can occur at any age, with cases documented in patients ranging from two to 85 years old, with a mean age of 28.5 years at presentation [1,2]. At presentation, 85-91% of patients are found to have localized disease, with the remainder showing evidence of local invasion or metastasis [2]. Primary tumors preferentially metastasize to the liver, portal vein, and spleen [1]. Surgical resection is the primary treatment for all stages of the disease. It is superior to chemotherapy, irradiation, radiofrequency ablation, transcatheter arterial chemoembolization, and transcatheter arterial infusion in a small patient sample with metastatic disease [3]. SPEN has a good prognosis, with a recent multicenter retrospective analysis of 118 patients showing a recurrence rate of 1.8% and a five-year survival rate of 97.7% at a median 59-month follow-up [4]. Studies have implicated progesterone in tumor pathogenesis and note that SPEN grows rapidly in pregnant women because of this [5-12]. While even pregnant patients with metastatic SPEN could have favorable outcomes, there may be significant barriers to traditional treatment that complicate management. We present the case of a young woman with a remote history of resected SPEN who presented with new liver metastasis during pregnancy. There is no established precedent for the management of such patients, and as such, different approaches were taken given the significant surgical limitations due to her tumor location and burden. We will discuss the patient's clinical course, their response to treatment, and our recommendations for managing similar cases.

## Case presentation

**Figure 1:**
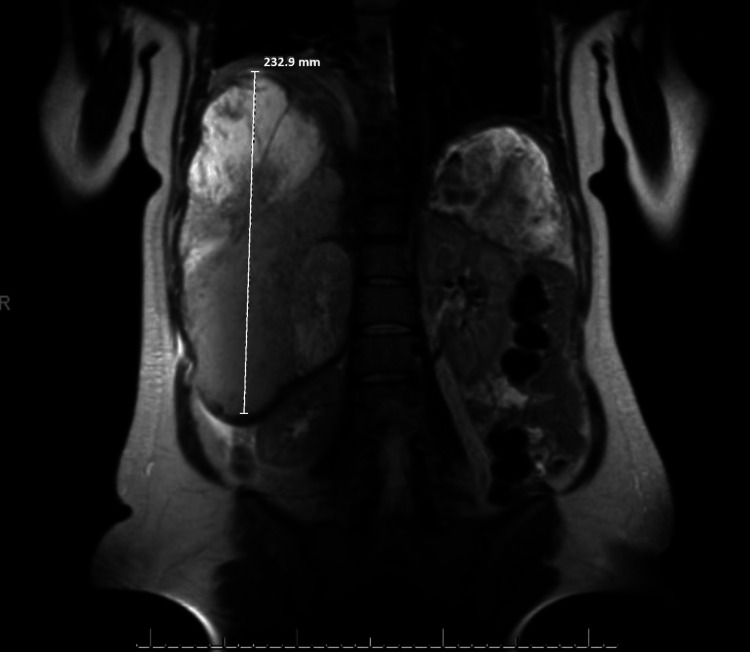
Coronal MRI of the abdomen and pelvis showing 23.3 cm (232.9 mm) cystic liver mass MRI: magnetic resonance imaging

**Figure 2:**
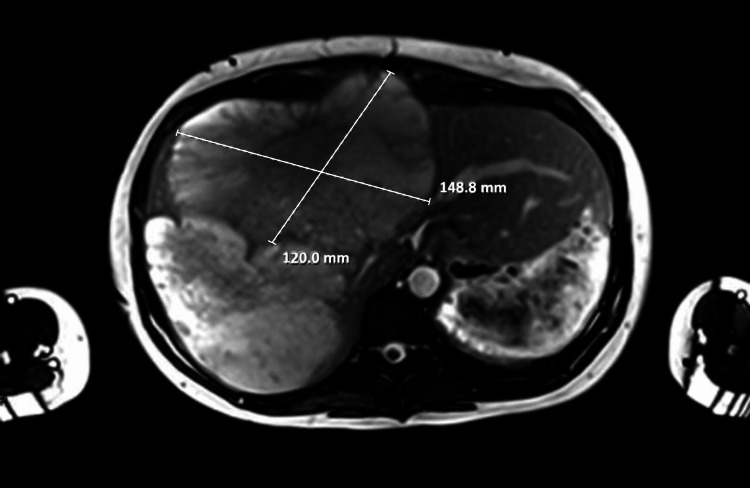
Sagittal MRI of the abdomen and pelvis showing 12 cm x 14.9 cm (120 mm x 148.8 mm) cystic liver mass alongside multiple small cystic masses MRI: magnetic resonance imaging

**Figure 3:**
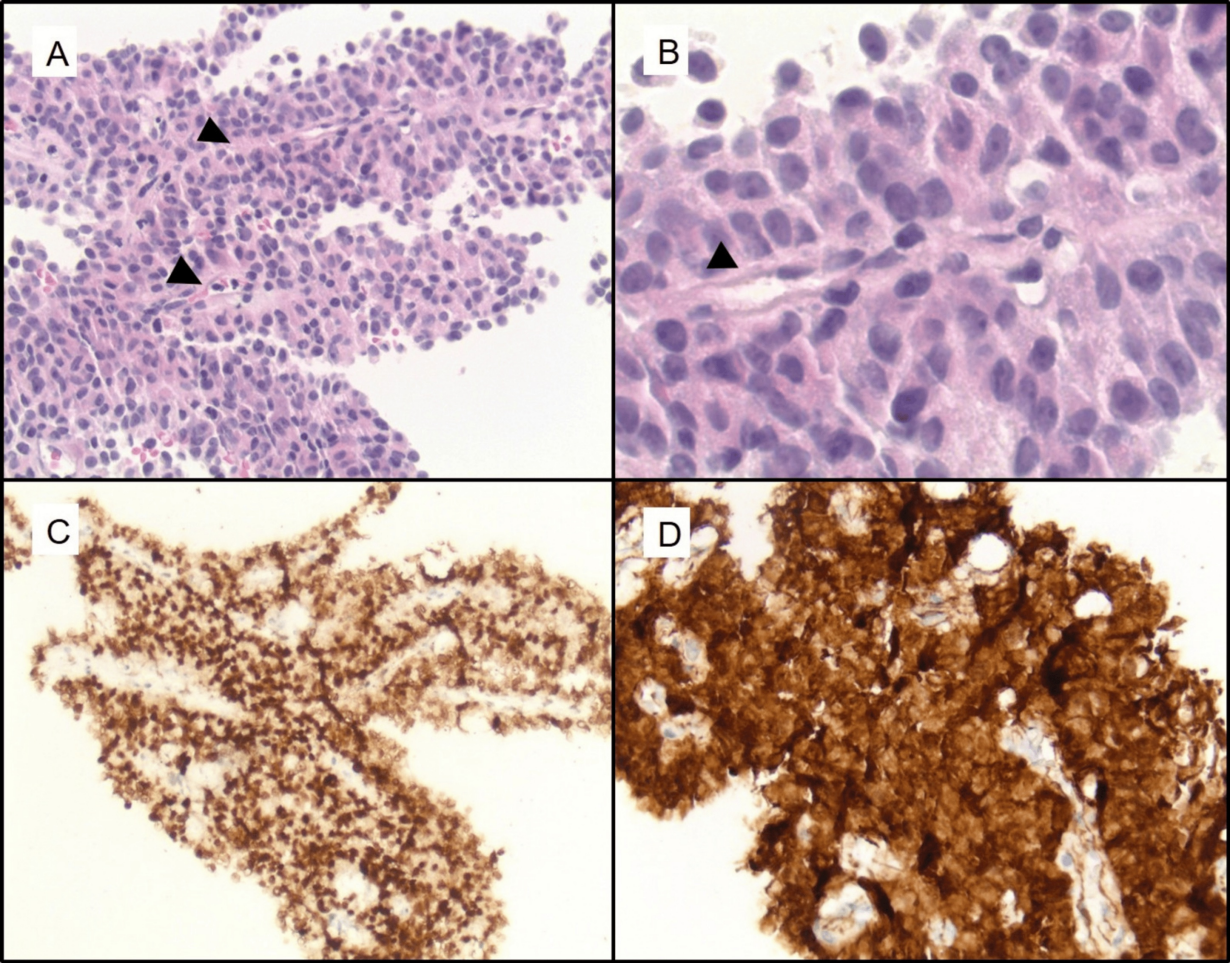
(A) H&E sections (20x) of the patient’s liver biopsy demonstrate fragments of tumor composed of monotonous, epithelioid tumor cells arranged around fibrovascular cores (arrowheads). (B) At higher magnification (40x), the tumor nuclei are round with minimal cytologic atypia and inconspicuous nucleoli. The endothelial spindle cells of the fibrovascular cores (arrowhead) are also seen. (C) Immunohistochemical stains demonstrate that the tumor demonstrates diffuse nuclear expression of LEF-1 with aberrant, non-membranous beta-catenin expression. (D) The tumor cells were also positive for CD10 and PR with weak cytokeratin expression (not pictured). H&E: hematoxylin and eosin, LEF-1: leukocyte enhancing factor, PR: progesterone receptor

A 36-year-old 22-week pregnant woman with a past medical history of early-stage SPEN status post partial pancreatectomy and splenectomy eight years prior presented to the emergency department with abdominal pain, shortness of breath, and tachycardia. On exam, the patient was tachycardic with a mildly distended abdomen consistent with being 22 weeks pregnant. Chest X-ray and CT showed an elevated right hemidiaphragm. The patient was referred to pulmonary medicine, which was concerned that symptoms were secondary to an enlarged liver causing compression of the lung and heart vasculature. Abdominal ultrasound showed diffuse, cystic, and solid liver lesions concerning for metastasis. Abdominal MRI confirmed the presence of many septated cystic masses measuring up to 12.5 x 11.6 x 23.3 cm and an unremarkable remnant pancreas (Figures 1-2). Liver biopsy confirmed a diagnosis of metastatic SPEN (Figure 3).

The patient was seen by oncology and started on gemcitabine for cytoreduction prior to delivery, given a significant tumor burden and related symptoms. She received two cycles of chemotherapy prior to delivering healthy twins at term through labor induction. Shortly after delivery, the patient was admitted for abdominal pain and found to have multiple bilateral pulmonary emboli, as well as extensive right common, internal, and external iliac vein deep vein thromboses. An abdominal CT scan during admission showed numerous heterogeneous, septated cystic masses in both liver lobes, which combined to span 24 x 34 x 20 cm. Hepatology, interventional radiology, and hepatobiliary surgery evaluated the patient and determined there was no evidence-based intervention they could offer.

Shortly after discharge, the patient underwent lattice radiation therapy. She was treated with 20 Gy in five fractions over seven days, for a total cumulative dose of 66.7 Gy. During that time, the patient was readmitted for recurrent right-sided pleural effusions and discharged with a PleurX catheter after cytology and infectious workup returned negative. Given worsening disease, second-line chemotherapy with FOLFIRINOX (5-fluorouracil, leucovorin, irinotecan, and oxaliplatin) was started and continued for eight cycles with evidence of continued disease progression on repeat imaging. Hormone therapy was suggested as the next line of treatment, given known tumor positivity for the progesterone receptor. The patient initially opted for oophorectomy rather than medical therapy; however, her gynecologist was not comfortable performing the procedure given her age and the absence of supportive evidence. Instead, she was planned to start goserelin, palbociclib, and anastrazole to treat her disease as a HER-/ER+/PR+ breast cancer based on a case report.

Shortly thereafter, the patient was admitted yet again for gastrointestinal bleeding and noted to have esophageal varices, portal hypertensive gastropathy, and duodenal ulceration on esophagogastroduodenoscopy. The tumor board evaluated her case after discharge and reinvolved hepatobiliary surgery and hepatology for possible living donor liver transplantation. During transplant workup, the patient received one dose of goserelin and anastrazole. Her insurance did not approve palbociclib. An abdominal CT one month later showed stable metastatic disease.

After a critical review of her case, hepatobiliary surgery changed course and took the patient for hepatic trisegmentectomy with sternotomy for suprahepatic inferior vena cava control, as well as partial right lung decortication. After a prolonged stay in the intensive care unit, the patient was discharged home and made a full recovery. She completed her surveillance period without disease recurrence. At this time, she is pregnant with her third child and disease-free.

## Discussion

SPEN is typically a slow-growing tumor that presents incidentally and is amenable to resection in most cases. Our patient presented with a significant metastatic tumor burden, causing dyspnea, deep vein thromboses, liver dysfunction, and portal hypertension. Given the extent of her disease, she was considered a poor surgical candidate, and limited evidence-based treatment options were available. Upon reviewing the literature, it is evident that some SPEN tumors are considered unresectable due to their invasive pattern or arterial encasement. In such cases, there are no guidelines to direct management. Radiation therapy, chemotherapy, and hormone therapy have been suggested in such situations [13,14].

Chemotherapy is successful in some patients, although there is limited evidence to support its use [3,14,15]. Radiation is not an option for the definitive treatment of SPEN at any stage of the disease. However, it has been shown to play a palliative role in metastatic disease in a small number of patients, reducing disease burden [3]. The low prevalence of SPEN and its slow-growing nature have made it difficult to establish guidelines for the treatment of patients who have progressed to advanced stages of disease. It is known that young women are disproportionately affected by the disease [1]. This predilection led to numerous studies in the twentieth century evaluating the role of sex hormones in tumor pathogenesis. Immunohistochemical studies consistently showed positivity for progesterone receptor but not for estrogen receptor. Data regarding the impact of sex hormones on tumor growth, progression, and prognosis were inconclusive; however, there is likely a role for progesterone in tumor pathogenesis [5-9]. This is supported by evidence of rapid tumor growth in pregnancy, a state of progesterone excess [10-12]. SPEN is particularly rare in pregnancy, with only six such cases documented in the literature. In all cases, the primary tumor was surgically resected during pregnancy, and patients underwent successful deliveries [10-12,16-18]. In one case, multiple resections were performed for metastatic disease with successful subsequent delivery [18].

Our patient's tumor was not amenable to surgery and did not respond to two lines of chemotherapy. With limited alternatives remaining and the patient's health in rapid decline, hormone therapy to decrease progesterone levels was suggested as a possible option. Surgical and medical hormone therapies were considered, but ultimately, medical therapy with goserelin, a gonadotropin-releasing hormone receptor agonist, and anastrazole, an aromatase inhibitor, was pursued. Repeat imaging after the first cycle of treatment showed stable disease. It is difficult to determine if the patient's disease would have remained stable, improved, or eventually progressed on continued hormone therapy, given that she underwent surgery shortly after starting it. This is one limitation of this study. However, considering her disease progressed during similar intervals on other therapies, additional investigation is warranted.

This case suggests that hormone therapy could be a viable option to prevent SPEN disease progression at the very least, which is an improvement from other options like chemotherapy and radiation. This is likely due to the slow-growing nature of the disease, which makes it a poor responder to therapies that target cell growth and division. Surgical resection will likely always remain the gold standard; however, in complex cases where surgical options are not immediately available, hormone therapy could serve as a bridge to more definitive management.

## Conclusions

While SPEN remains a very rare malignancy with generally favorable outcomes, it is important to recognize that advanced cases, which are most likely to affect young, fertile women, could be devastating. This case serves as a reminder that surgical resection of SPEN, even in metastatic disease, remains the gold standard for the time being. It also suggests that in cases where surgical management is not an immediate option, hormone therapy may provide greater benefit than chemotherapy and radiation and may play a role as a bridge to more definitive therapy.
